# Effect of arm movement on balance performance in children: role of expertise in gymnastics

**DOI:** 10.1186/s13104-022-06182-1

**Published:** 2022-12-09

**Authors:** Thomas Muehlbauer, Joana Heise, Mathew W. Hill

**Affiliations:** 1 Division of Movement and Training Sciences/Biomechanics of Sport, University of Duisburg-Essen, Gladbecker Str. 182, 45141 Essen, Germany; 2 Center for Sport, Exercise and Life Sciences, School of Life Sciences, Coventry University, Warwickshire, UK

**Keywords:** Postural control, Standing, Walking, Reaching, Sports expertise

## Abstract

**Objective:**

Studies have shown that balance performance is better in gymnasts compared to age-/sex-matched controls and further studies revealed superior performance when arms were free to move during assessment of balance. However, it is unknown whether free arm movement during balance testing differentially affects balance performance with respect to sports expertise (i.e., gymnasts are less affected than age-/sex-matched controls). Therefore, we investigated the effect of arm movement on balance performance in young female gymnasts compared to age-/sex-matched controls while performing balance tasks with various difficulty levels.

**Results:**

In both samples, balance performance (except for the timed one-legged stance) was significantly better during free compared to restricted arm movement conditions and this was especially observed in the highest task difficulty condition of the 3-m beam walking backward test. These findings revealed that balance performance is positively affected by free arm movements, but this does not seem to be additionally influenced by the achieved expertise level in young gymnasts.

## Introduction

Previous studies showed that arm movements play an important role for static and dynamic postural control [1, 2]. Specifically, balance performance was better under free (i.e., arms were moved freely in all directions) compared to restricted (i.e., hands were placed on the hips or crossed over the chest) arm movement conditions. Further studies suggest that the positive effect of free arm movement on postural control is a relatively robust finding that occurs independently of participants’ age [3] as well as the type [2] and difficulty/complexity level [1, 4–6] of the tested balance task.

While the aforementioned factors have been relatively well studied, the influence of arm movement on balance performance with respect to sports expertise has received less attention. This is surprising because, for example, in sports such as dancing, figure skating and gymnastics, a high level of postural control is required to be successful [7]. In fact, even young gymnasts showed shorter and slower postural sway [8, 9] as well as less time for balance recovery [9] compared to untrained age-/sex-matched controls, which suggests that the positive effect of arm movement on postural control might be less pronounced in gymnasts than in controls.

Thus, the present study investigated whether the positive effect of free arm movement on balance performance is also evident in individuals who trained their postural control over several years compared to untrained age-/sex-matched controls. We hypothesized that the effect of arm movement will be detected in both but less in gymnasts (due to their already well-developed postural control system [8, 9]) than in untrained age-/sex-matched controls. Further, we assumed that the difference between free compared to restricted arm movement conditions will increase as the difficulty level of the balance task increases, especially for the untrained the controls.

## Methods

### Participants

Thirty-six children (18 female gymnasts, 18 untrained, age-matched girls) participated in this study (Table 1). There were no significant differences in the participants' characteristics. All participants were healthy and free of any neurological or musculoskeletal impairments. None of the participants had prior experience with the performed balance tests. Written informed consent and subject’s assent were obtained from all participants before the start of the study. In addition, parent’s approval was obtained for minors.

**Table 1:** Characteristics of the study participants by group (*N* = 36)

Characteristic	Gymnasts (*n* = 18)	Controls (*n* = 18)	*p*-value
Age [years]	10.9 ± 0.9	11.3 ± 0.5	0.127
Maturity offset^a^ [years from PHV]	− 0.8 ± 1.1	− 0.5 ± 0.5	0.263
Training experience [years]	3.2 ± 2.5 (range: 1–8)	–	–
Body mass [kg]	39.1 ± 11.3	41.1 ± 8.7	0.563
Body height [cm]	147.8 ± 13.2	150.5 ± 6.1	0.439
Body mass index [kg/m^2^]	17.6 ± 2.3	18.1 ± 3.2	0.600
Leg length [cm]	76.7 ± 9.5	77.3 ± 6.5	0.830

Data are presented as group mean values ± standard deviations

^a^Maturity offset was calculated as years from peak height velocity (PHV) by using the formula provided by Moore et al. [18]

### Assessment of balance

Balance was assessed by using the timed One-Legged Stance (OLS) test. Participants were asked to stand without shoes on their dominant kicking leg (determined by self-report). The participants were instructed to stand for as long as possible but for a maximum of 60 s. The OLS was conducted under three different conditions with increasing level of task difficulty: (1) with eyes closed on firm ground (EC, FI); (2) with eyes opened on foam (i.e., AIREX Balance-pad) ground (EO, FO); (3) with eyes closed on foam ground (EC, FO). One practice trial followed by one data-collection trial was executed and the maximal stance time (s) was used for further analysis. The OLS test is a valid (concurrent and discriminative) and reliable (moderate to excellent) test of balance performance in children [10, 11].

Further, balance was assessed by means of the 3-m beam walking backward test. Specifically, three wooden beams (length: 3 m; height: 5 cm) that differed in width (i.e., 6, 4.5, and 3 cm) were used. The participants were asked to walk at a self-selected speed backward from the start to the end of the beams but for a minimum of eight steps. One practice trial followed by 2 data-collection trials were performed. The number of steps for the 2 data-collection trials per beam width was summed, which resulted in a maximum of 16 steps per beam width and 48 steps in total.

Finally, balance was determined using the Lower Quarter Y-Balance (YBT-LQ) test kit. The test kit comprised a central footplate to which three pipes were attached in the anterior (AT), posteromedial (PM), and posterolateral (PL) directions. Each pipe is equipped with a moveable reach indicator block and marked in 1.0-cm increments. While standing with the dominant leg on the central footplate, the participants were instructed to reach with the non-dominant leg as far as possible in the AT, PM, and PL directions. The absolute maximal reach distance (cm) per reach direction was noted. Three practice trials followed by three data-collection trials were executed. Before testing, the length (cm) of the dominant leg for each participant was determined from the anterior superior iliac spine to the most distal aspect of the medial malleolus [12]. Thereafter, the normalized maximal reach distance (% leg length [LL]) per reach direction was computed by dividing the absolute maximal reach distance by LL and then multiplying by 100. Further, the normalized (% LL) composite score (CS) was calculated as the sum of the absolute maximal reach distance per reach direction divided by three times LL and then multiplied by 100. All balance tests were conducted under two conditions: free (i.e., arms were moved freely in all directions) and restricted (i.e., hands were placed on the hips) arm movements.

### Statistical analyses

Descriptive data were reported as group means ± standard deviations. Assumptions of normality (Shapiro–Wilk Test) and homogeneity of variance/sphericity (Mauchly Test) were checked and confirmed prior to conducting parametric analyses. For the OLS and the 3-m beam walking backward test, an arm × expertise × task difficulty repeated measures analysis of variance (ANOVA) was conducted. Further, an arm × expertise repeated measures ANOVA was performed for the YBT-LQ test. When significant differences occurred, Bonferroni-adjusted post-hoc tests (i.e., paired *t*-tests) were performed. Further, effect size (*η*_p_^2^) was calculated and reported as small (0.02 ≤ *η*_p_^2^ ≤ 0.12), medium (0.13 ≤ *η*_p_^2^ ≤ 0.25), and large (*η*_p_^2^ ≥ 0.26) [13]. All statistical analyses were conducted using Statistical Package for Social Sciences version 27.0 and the α-value was a priori set at *p* < 0.05 for all comparisons.

## Results

Table 2 displays balance performance with free compared to restricted arm movement by expertise and Table 3 shows the main and interaction effects of the repeated measures ANOVA per outcome. A main effect of arm was observed for two out of three conditions (except for the 6-cm wide beam) of the 3-m beam walking backward test, for two out of three reach directions (except for the PM reach direction) as well as the CS of the YBT-LQ test but not for any condition of the OLS test. Post-hoc analyses revealed that performance was significantly better during free compared to restricted arm movement.

**Table 2:** Balance performance during free compared to restricted arm movement conditions by group

Test/outcome	Gymnasts (*n* = 18)		Controls (*n* = 18)	
	Free	Restricted	Free	Restricted
OLS				
OLS time; EC, FI [sec]	29.8 ± 20.2	26.9 ± 18.0	31.1 ± 23.4	33.6 ± 24.9
OLS time; EO, FO [sec]	43.1 ± 19.1	39.9 ± 22.6	39.2 ± 22.9	33.2 ± 22.5
OLS time; EC, FO [sec]	12.7 ± 15.9	7.1 ± 9.2	7.9 ± 5.6	5.1 ± 5.1
3-m beam walk				
6.0-cm beam walk [steps]	14.8 ± 2.7	14.9 ± 2.6	13.7 ± 4.0	12.0 ± 4.3
4.5-cm beam walk [steps]	12.9 ± 4.9	11.3 ± 4.0	12.9 ± 4.0	10.2 ± 3.9
3.0-cm beam walk [steps]	9.8 ± 4.9	5.6 ± 2.8	7.7 ± 3.5	5.3 ± 3.3
YBT-LQ				
YBT-LQ: AT reach [% LL]	110.7 ± 20.7	102.4 ± 20.0	78.8 ± 9.2	75.3 ± 8.9
YBT-LQ: PM reach [% LL]	140.7 ± 18.1	139.3 ± 16.5	111.5 ± 16.5	105.7 ± 11.5
YBT-LQ: PL reach [% LL]	134.4 ± 18.6	129.8 ± 15.7	108.7 ± 14.2	103.5 ± 12.4
YBT-LQ: CS [% LL]	128.6 ± 15.8	123.8 ± 13.5	99.7 ± 12.3	94.9 ± 9.7

Data are presented as group mean values ± standard deviations

*AT *  Anterior; *CS* Composite score; *EC* Eyes closed; *EO* Eyes opened; *FI* Firm ground; *FO* Foam ground; *LL* Leg length; *OLS* One-Legged Stance test; *PL* posterolateral; *PM* Posteromedial; *YBT-LQ* Lower Quarter Y Balance test

**Table 3:** Main and interaction effects of the repeated measures ANOVA per outcome measure

Test/Outcome	Main effects		Interaction effects	
	Arm	Expertise	Arm × expertise	Arm × expertise × task difficulty
OLS				
OLS time; EC, FI [sec]	0.946 (0.01)	0.546 (0.01)	0.413 (0.02)	0.131 (0.05)
OLS time; EO, FO [sec]	0.194 (0.05)	0.414 (0.02)	0.684 (0.01)	
OLS time; EC, FO [sec]	0.063 (0.10)	0.177 (0.05)	0.520 (0.01)	
3-m beam walk				
6.0-cm beam walk [steps]	0.216 (0.05)	0.043 (0.12)^b^	0.159 (0.06)	0.028 (0.10)
4.5-cm beam walk [steps]	0.001 (0.27)^a^	0.646 (0.01)	0.349 (0.03)	
3.0-cm beam walk [steps]	< 0.001 (0.47)^a^	0.286 (0.03)	0.133 (0.07)	
YBT-LQ				
YBT-LQ: AT reach [% LL]	0.025 (0.14)^a^	< 0.001 (0.55)^b^	.349 (0.03)	–
YBT-LQ: PM reach [% LL]	0.075 (0.09)	< 0.001 (0.55)^b^	.271 (0.04)	–
YBT-LQ: PL reach [% LL]	0.018 (0.15)^a^	< 0.001 (0.47)^b^	.882 (0.01)	–
YBT-LQ: CS [% LL]	0.007 (0.19)^a^	< 0.001 (0.61)^b^	.982 (0.01)	–

Values are *p*-values and effect sizes (*η*_p_^2^) in brackets. .02 ≤ *η*_p_^2^ ≤ .12 indicates small, .13 ≤ *η*_p_^2^ ≤ .25 indicates medium, and *η*_p_^2^ ≥ .26 indicates large effects

*AT* Anterior; *CS* Composite score; *EC* Eyes closed; *EO* Eyes opened; *FI* Firm ground; *FO* Foam ground; *LL* Leg length; *OLS* One-Legged Stance test; *PL* Posterolateral; *PM* Posteromedial; *YBT-LQ* Lower Quarter Y Balance test

^a^Indicates a significant (*p* < .05) performance difference between free compared to restricted arm movement condition.

^b^Indicates a significant (*p* < .05) difference between gymnasts and untrained age-/sex-matched controls.

Further, a main effect of expertise was obtained for the least difficulty walking condition (i.e., 6-cm beam width), for all reach directions and the CS of the YBT-LQ test but again not for any condition of the OLS test. Post-hoc analyses indicated that performance was significantly better in gymnasts compared to controls.

Additionally, the arm × expertise × task difficulty interaction reached the level of significance for the 3-m beam walk test only. Post-hoc analyses showed differences in favor of the free arm movement conditions for the controls regardless of the task difficulty level, but this result emerged for the gymnasts only for the most difficult condition (i.e., 3-cm beam width). Lastly, the arm × expertise interaction did not reach the level of significance.

## Discussion

The present study investigated the effect of arm movement on balance performance in young female gymnasts compared to untrained age-/sex-matched controls. For the 3-m beam walking backward test (except while walking on the 6-cm wide beam) and the YBT-LQ test (except for the PM reach direction) but not for the OLS test, we observed significantly better balance performance under free compared to restricted arm movement conditions. This finding is partially in accordance with our first hypothesis and corresponds with those from previous studies that also found a positive effect of free arm movement on balance performance. For example, Objero et al. [1] reported less postural sway during standing with free versus restricted arm movement. Additionally, Hill et al. [2] reported shorter times to walk a limited distance of two meters while arm movements were free to use. Lastly, Hébert-Losier et al. [14] detected greater YBT-LQ reach distances when arms could freely move. From a practitioner's perspective, it can therefore deduce that free arm movement should be allowed if the goal is to detect greater balance performances. The observed lack of arm movement effects on OLS performance can most likely be explained by “ceiling effects”. Precisely, this task is representative of steady-state static balance control, where the center of mass can relatively easily be held over the base of support [15]. Thus, arm movements have no additional positive influence on stance duration.

The female gymnasts outperformed the untrained age-/sex-matched controls with respect to the 3-m beam walking backward test (i.e., while walking on the 6-cm wide beam) and the YBT-LQ test (i.e., all reach directions and the CS). These findings are in line with previous studies [8, 9] that reported better balance performance in gymnasts compared to untrained controls. For example, Busquets et al. [9] investigated young gymnasts (mean age: 9.7 ± 1.1 years) and age-matched controls that were asked to stand quietly on a force plate. They detected shorter postural sway and less time to recover initial balance for the gymnasts compared to the controls. Most likely, years of training and related adaptation processes of the vestibular, visual, and somatosensory systems [16, 17] are responsible for the better balance performance in gymnasts compared to the controls. Although the gymnasts performed better than the controls, the positive effect of arm movement was present in both groups (i.e., occurred independent of sports expertise) as indicated by the non-significant arm × expertise interactions. Therefore, this factor, but also age [3] as well as the type [2] and difficulty/complexity level [1, 4–6] of the balance task, did not seem to have an additional moderating impact.

Partially in line with our second hypothesis and as indicated by the arm × expertise × task difficulty interaction, the difference between the two test conditions increased as the beam width for the 3-m beam walk decreased, and this was especially observed for the smallest beam width. This finding is in accordance with former studies [1, 5, 6] that reported a greater difference between free compared to restricted arm movement conditions in more compared to less difficult postural tasks. For example Boström et al. [5] examined young adults while walking forward over three beams of varying width. They reported that movements of the upper body significantly increased when the beam width decreased—as in the present study—from 6 cm over 4.5 cm to 3 cm. However, the above-mentioned interaction was only found for the 3-m beam walking backward test but not for the OLS test. Again, “ceiling effects” as already stated could in turn be responsible for this result.

## Conclusion

The present study compared the effect of arm movement on balance performance between female gymnasts and untrained age-/sex-matched controls that performed balance tasks with increasing difficulty levels. In both samples, free compared to restricted arm movements resulted in significantly better balance performances and this was more obvious in more difficult tasks. Thus, balance assessment while using arm movements facilitates performance, especially in tasks with a high difficulty level but this seems to be unaffected by the achieved expertise level in young gymnasts (i.e., children). Therefore, future studies should examine whether more experienced gymnasts (i.e., adult or master) will better use arm movements for postural control.

## Limitations

- Only young female gymnasts versus untrained age-/sex-matched controls were investigated, which prevents the transfer of our findings to male subjects and older age groups (i.e., adolescents or adults).
- No instrumented biomechanical measurements (e.g., using a force plate) but frequently used field tests were applied.
- No kinematic (e.g., using motion capture) but performance data (i.e., stance duration, number of steps, reach distance) were analyzed.

## Data Availability

The data generated and analyzed during the present study are not publicly available due to ethical restrictions but are available from the corresponding author upon reasonable request.

## Abbreviations

ANOVA: Analysis of variance
AT: Anterior
CS: Composite score
EC: Eyes closed
EO: Eyes opened
FI: Firm ground
FO: Foam ground
LL: Leg length
OLS: One-legged Stance test
PL: Posterolateral
PM: Posteromedial
YBT-LQ: Lower Quarter Y Balance test
